# Production and Characterization of Collagen-Based Film with Natural Oil and Zinc Oxide

**DOI:** 10.1021/acsomega.5c13675

**Published:** 2026-04-14

**Authors:** Hilal Cikot, Bugra Ocak, Gulsah Turkmen

**Affiliations:** † Graduate School of Natural and Applied Sciences, Materials Science and Engineering, 37509 Ege University, 35100 Bornova, Izmir, Turkiye; ‡ Faculty of Engineering, Department of Leather Engineering, Ege University 35100 Bornova, Izmir, Turkiye

## Abstract

The leather sector generates substantial quantities of protein-rich residues, and converting these residues into value-added products is crucial for sustainability, environmental and human health, and the circular economy. For this purpose, collagen hydrolysate (CH) was produced from trimming residues of the leather sector via bromelain-mediated enzymatic hydrolysis, and films incorporating nano zinc oxide (ZnO) and *Calendula officinalis* L. (CO) essential oil were fabricated and subsequently characterized. In this study, prior to enhancing the functional properties of CH-based films, one of their primary limitations—water barrier performance—was addressed by producing CH/ZnO films containing 0–4% ZnO nanoparticles, identifying CH/ZnO^2%^ as the optimal formulation; at this fixed ratio, CH/ZnO^2%^ films were enriched with CO essential oil at 2%, 4%, 6%, and 8% to prepare CH/ZnO/CO films, which were subsequently characterized. In general, the results show that CH/ZnO/CO-based environmentally friendly and sustainable films obtained from leather industry trimming wastes are promising in terms of applicability to packaging materials.

## Graphical abstract

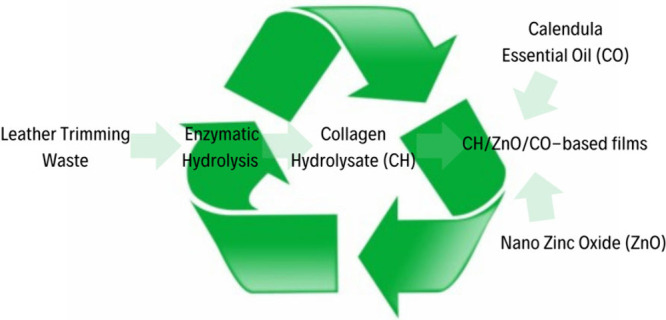

## 1 Introduction

In the contemporary global economy, the leather industry constitutes a strategically significant sector within international trade, driven by escalating worldwide demand for leather and leather-based products, while simultaneously serving as a major source of employment through extensive value chains encompassing raw material processing, manufacturing, and export-oriented production.^1,2^ Nevertheless, the substantial pollution loads generated by industrial operations have led to the classification of the leather industry among high-polluting industrial sectors due to the intensive discharge of chemically complex effluents and solid wastes throughout its production processes.^1,3,4^ The improper management of tannery-derived solid wastes, generated by an industry producing approximately 23 billion square meters of leather annually and occupying a vital position in the industrial economy, represents one of the most critical environmental challenges to achieving sustainability, particularly in developing countries.^2,5^ During the conversion of one ton of raw hides, roughly 200–250 kg of finished leather is produced, leading to the creation of considerable amounts of solid and liquid byproducts throughout the manufacturing cycle.^1^ According to statistical data, the conversion of one ton of raw hides produces approximately 40–50 m^3^ of effluent and 750–800 kg of solid residues, highlighting the resource- and waste-intensive characteristics of tannery operations.^1,2^ Given that approximately 600,000 tons of leather-related solid waste are generated globally each year, mitigating the adverse environmental impacts of the leather industry has become an increasingly critical priority.^1,2^ In many cases, these wastes are partially utilized through low-cost, nonscientific practices or disposed of in landfills, and the improper management of tannery solid wastes constitutes one of the most significant environmental challenges to achieving sustainability, particularly in developing countries.^1,2^ As inadequate solid waste management strategies exert detrimental effects on the environment and compromise human health within and around tannery facilities, the valorization of industrial wastes into viable materials represents a challenging yet critical task in the context of circular economy implementation and environmental protection.^2,4^ Given that the molecular structure of leather is fundamentally based on collagen proteins, leather industry wastes contain substantial amounts of collagen-derived proteins, rendering them a potentially valuable secondary raw material for diverse industrial and biotechnological applications.^1,2,4^ Statistical data indicate that approximately 418,000 tons of raw trimming waste are generated globally each year and that these wastes are not adequately utilized; consequently, the comprehensive and efficient valorization of raw trimming wastes has become increasingly important.^6^

Fossil-fuel-derived synthetic polymers find applications in medical, agricultural, and packaging sectors under various environmental and public health limitations; however, extensive utilization of petroleum-derived commercial films in the packaging industry has resulted in significant global environmental issues.^7,8^ The escalating impacts of pollution-driven global warming and the exhaustion of finite resources have made the urgent adoption of eco-friendly plastics essential; consequently, numerous countries and regions are encouraging the fabrication and application of packaging materials derived from renewable polymers while simultaneously limiting or banning disposable, petroleum-based films that are nonbiodegradable and whose production and disposal pose environmental and public health hazards.^8,9^

The environmental and health issues linked to petroleum-derived packaging have increased focus on developing biodegradable packaging using eco-friendly biopolymers, including proteins, carbohydrates, and lipids.^8,10^ Biopolymer-based packaging films exhibit limited industrial applicability compared with synthetic polymer-based counterparts due to their inherently inferior mechanical strength, thermal stability, and moisture barrier properties.^10^ Recently, the fabrication of films composed of biopolymers sourced from safe and sustainable biomolecules as substitutes for synthetic polymers in packaging has gained increasing interest due to their degradability, safety, renewability, and broad accessibility.^10,11^ While films derived from these biopolymers are unlikely to fully substitute conventional synthetic polymer-based packaging, they are expected to satisfy contemporary consumer preferences for eco-friendly, safe, and naturally sourced products.^8^ Several studies have further increased interest in biodegradable films by highlighting the potential for natural biopolymeric materials to be used in combination with synthetic materials, thereby enabling the development of hybrid systems with improved functional performance.

Among films based on biopolymeric structures, polysaccharide- or protein-derived films generally exhibit favorable mechanical and organoleptic properties; however, they are inherently sensitive to moisture. Conversely, lipid-based films, while exhibiting excellent water vapor barrier properties, may be opaque and brittle, limiting their practical applicability. Protein-based films exhibit high water and moisture absorption and are characterized by low water vapor permeability (WVP). Therefore, a more efficient moisture barrier is needed to reduce the impact of water in foods, maintain low water activity, and inhibit reactions that cause spoilage. Various strategies have been utilized to enhance the water barrier performance of films derived from proteins. To this end, efforts have been made to address these limitations by developing composite films through the blending of different types of polymers. In addition, strategies such as the incorporation of hydrophobic agents or the cross-linking of protein chains have also been applied to enhance film performance.^12,13^

Collagen, which can be easily processed into mechanically robust films, undergoes hydrolysis—acidic, alkaline, or enzymatic—to form gelatin, and further hydrolysis produces collagen hydrolysate (CH), a mixture of peptides of varying molecular weights.^14^ Nevertheless, the structural and functional characteristics of pure CH films still need improvement to satisfy the demands of various application contexts. The design of functional composite materials to improve CH-based film performance and expand their use in packaging has garnered significant research attention in recent years.^8^

Zinc oxide (ZnO) nanoparticles have attracted considerable research interest due to their biocompatibility, low production cost, scalability, strong UV absorption, thermal and chemical stability, and notable photocatalytic and antibacterial activities, making them an economically advantageous alternative to other inorganic agents and widely investigated for enhancing the functional attributes of biopolymer-based films for packaging applications.^10,15–17^ Recognized as GRAS and approved by the U.S. Food and Drug Administration (FDA), zinc oxide nanoparticles are considered safe for human health, do not accumulate in tissues, are mainly excreted from the body, and effectively extend the microbiological and chemical shelf life of packaged foods without adversely affecting organoleptic properties.^18,19^ Regarding European Food Safety Authority (EFSA) regulations, the use of zinc oxide in food-contact packaging has raised safety concerns based on toxicological data, with EFSA establishing a tolerable upper intake level of 25 mg day^–1^, while the FDA permits a higher limit of 40 mg day^–1^.^20^ Due to their tendency to agglomerate and disperse poorly in the film matrix, ZnO nanoparticles can reduce film performance unless nanoparticle loading is optimized to prevent aggregation and ensure uniform dispersion, which is essential for maintaining the functionality and processing efficiency of sustainable nanocomposite packaging.^9,21,22^

Essential oils are volatile, naturally occurring organic compounds generated via the secondary metabolic pathways of aromatic plants and are extensively acknowledged for their broad-spectrum bioactivities, including antibacterial, antifungal, and antioxidant effects,^8,23^ which are primarily attributed to bioactive constituents such as aldehydes, phenols, esters, terpenes, and other compounds.^8^ Recently, adding natural essential oils to pure films has been demonstrated to improve both their antioxidant and antibacterial activities as well as their water vapor barrier performance and water solubility (WS, %).^23^ *Calendula officinalis* L. (CO), widely referred to as marigold and a member of the Asteraceae family, is a yearly herb cultivated globally for ornamental and medicinal uses, exhibiting antioxidant, anti-inflammatory, and antimicrobial activities attributable to its carotenoids, terpenoids, terpenes, flavonoids, quinones, coumarins, and other bioactive constituents.^24–26^ Due to these properties, CO has found applications not only in traditional medicine but also in pharmaceutical formulations and food products.^26^

A review of the literature reveals that, as a novel approach, the use of essential oil and nanoparticle combinations to enhance the properties of CH-based films derived from leather industry trimming wastes has not yet been reported. In the study, CH was initially obtained from leather industry trimming wastes, and its characterization was subsequently performed. In the second phase, CH-based films were prepared by incorporating ZnO nanoparticles at different concentrations (0–4%) to reduce WS (%), a limitation of CH films, and the resulting films were evaluated for thickness, moisture content (MC, %), WS (%), and WVP. After identifying the CH/ZnO film formulation exhibiting the optimal water barrier performance, the films were further enriched with varying concentrations of CO essential oil to produce CH/ZnO/CO-based films, which were subsequently characterized in terms of thickness, MC (%), WS (%), WVP, tensile strength (TS), elongation at break (EAB, %), elastic modulus (EM), color, light transmittance, and analyzed using Fourier Transform Infrared Spectroscopy (FTIR), Scanning Electron Microscopy (SEM), and Differential Scanning Calorimetry (DSC).

## 2 Materials and Methods

### 2.1 Materials

Cattle leather trimming waste were collected from a tanning plant located in Manisa, stored in polyethylene containers with ice, and delivered to the laboratory. Calcium hydroxide (Ca­(OH)_2_, ≥95.0%, molecular weight: 74.09), hydrochloric acid (HCl), bromelain from pineapple stem (≥3 units/mg protein), citric acid (≥99.5%), glycerol (≥99.5%), nano ZnO (<100 nm particle size), Tween 80, Coomassie Brilliant Blue R-250, 2,2-diphenyl-1-picrylhydrazyl (DPPH), 2,2′-azino-bis­(3-ethylbenzothiazoline-6-sulfonic acid) diammonium salt (ABTS), iron (powder, 97%), sodium chloride (≥99.0%), activated carbon, and protein markers (10–225 kDa) were obtained from Sigma-Aldrich, while CO essential oil was procured from Farmasi Dr. C. Tuna.

### 2.2 Methods

#### 2.2.1 Preparation of Trimming Wastes

Bovine leather trimming wastes were transported to the laboratory from Manisa in ice-filled containers. After hairs and fats were removed from the trimming wastes, they were thoroughly washed with tap water and stored at low temperature (−20 °C). Prior to analysis, the frozen trimming wastes were thawed overnight at 4 °C, thoroughly rinsed under running tap water to remove surface contaminants and blood, and then allowed to drain and air-dry.

#### 2.2.2 Extraction and Yield of CH from Hide Trimming Wastes

As a pretreatment step, noncollagenous materials were removed from the trimming waste by soaking the samples in 0.2 M Ca­(OH)_2_ solution at a waste-to-solution ratio of 1:5 (w/v), under ambient conditions maintained at 25 ± 1 °C, for 3 h, with the solution renewed every 2 h. Subsequently, the alkali-treated trimming wastes were repeatedly rinsed with distilled water until a neutral pH was achieved in the wash effluent. The trimming wastes were cut into approximately 2 × 1 cm^2^ pieces and washed thoroughly again. The trimming wastes were subsequently swollen by continuous stirring at ambient temperature in a 1% HCl solution using a waste-to-solution ratio of 1:10 (w/v) for 20 h, followed by repeated rinsing with distilled water until the effluent reached neutral pH.

For enzyme solution preparation, distilled water was brought to pH 4.6, and bromelain (0.3 g/L) was dissolved in a 0.1 M citric acid medium. The swollen trimming waste samples were enzymatically processed for 5 h using a bromelain solution (0.3 g/L) prepared in a 0.1 M citric acid medium, at a sample-to-solution ratio of 1:6 (w/v) and under the supplier-recommended optimum conditions (pH 4.6, 25 °C). To inactivate bromelain, the mixture was subjected to thermal treatment in a water bath at 90 °C for 15 min, followed by immediate cooling to ambient temperature using an ice bath. Subsequently, the collagen-containing solution was subjected to centrifugation at 12,800 × *g* for a duration of 20 min. Following centrifugation, the collected supernatant was lyophilized, and the resulting CH powder was preserved in sealed containers for subsequent analyses.

#### 2.2.3 Electrophoretic Analysis of CH

Sodium dodecyl sulfate polyacrylamide gel electrophoresis (SDS-PAGE) under denaturing conditions was employed to evaluate the molecular weight profile of the obtained CH. The proteolyzed trimming waste was combined with the loading buffer at a mass ratio of one part sample to two parts buffer. Following incubation in a water bath at 95 °C for 5 min, the samples were applied to polyacrylamide gels consisting of a 12% resolving layer and a 4% stacking layer. Thereafter, electrophoresis was conducted under a steady current of 25 mA for each gel until the bromophenol blue tracking dye migrated to the gel’s lower edge. The gel was subsequently stained with 0.1% Coomassie Brilliant Blue for 2 h and destained in a solution comprising 30% (w/w) methanol and 10% (w/w) acetic acid, while a molecular weight marker spanning 10–225 kDa was used to assess the molecular weight distribution of the enzymatically hydrolyzed trimming residues.

#### 2.2.4 Preparation of CH/ZnO-Based Films Containing CO

CH-based films were prepared using the solution casting technique, in which CH (5% w/v) was dispersed in deionized water and stirred at 50 °C until a homogeneous solution was obtained. Glycerol was subsequently incorporated into the CH solution as a plasticizing agent at a level corresponding to 25% of the CH mass. ZnO nanoparticles were dispersed in 100 mL of the CH solution in water and subjected to ultrasonication for 30 min prior to application. ZnO nanoparticle dispersions were then added to the film solutions at 0.5%, 1.0%, 2.0%, and 4.0% of the CH weight, and stirring was continued until homogeneous solutions were obtained. The resulting films were designated according to the ZnO nanoparticle concentration as CH, CH/ZnO^0.5%^, CH/ZnO^1.0%^, CH/ZnO^2.0%^, and CH/ZnO^4.0%^, respectively.

Subsequently, CO essential oil was incorporated at levels of 2.0%, 4.0%, 6.0%, and 8.0% (w/w), with Tween 80 included as the emulsifying agent in an amount equivalent to the essential oil content. The solutions were agitated at 150 rpm until the attainment of homogeneous mixtures. Finally, the solutions were poured into polyethylene molds and left to dry under room conditions for a period of 48 h to enable film formation. Subsequently, the films were carefully removed from the molds and equilibrated at 25 °C under 50% relative humidity for a further 48 h prior to testing.

#### 2.2.5 Film Thickness and Mechanical Properties

The film thickness was determined using a digital micrometer with a measurement accuracy of 0.001 mm. For each test sample, the thickness at 10 randomly selected points was measured, and the mean value was calculated.

The TS, EAB (%), and EM of the CH/ZnO^2%^ and CH/ZnO^2%^/CO films were determined using a texture analyzer (TA.XT2i, Stable Micro Systems, Surrey, UK). Film specimens were cut into strips with an average dimension of approximately 10 mm × 55 mm for tensile testing. The tensile tests were conducted under averaged conditions, with a crosshead/test speed of 30 mm/min, an initial force of 0.1 N, a stretching distance of 40 mm, and a testing temperature of 25 °C. Each sample was evaluated multiple times, with an average of eight replicate measurements performed per sample to ensure reliable and accurate results.

#### 2.2.6 MC (%)

A weight-based approach was employed to determine the MC (%) of CH/ZnO^2%^ and CH/ZnO^2%^/CO-based film samples containing different concentrations of CO essential oil.

Film strips (3 × 4 cm^2^) were weighed before (*m*_i_) and after drying (*m*_f_) to constant mass in a laboratory oven at 60 ± 1 °C, and the MC (%) was calculated using eq 1 provided.

1 $$MC\;\left(\%\right)=\frac{m_{i}-m_{f}}{m_{i}}\times 100$$

#### 2.2.7 WS (%)

Each film specimen was sectioned into 2 × 3 cm^2^ segments and conditioned in a CaCl_2_-filled desiccator at 0% relative humidity for 24 h (*m*_i_). The specimens were then submerged in 85 mL of distilled water maintained at 25 °C and agitated for 1 h using a magnetic stirrer. The remaining residues were recovered by filtration through Whatman No. 1 filter paper, then oven-dried at 60 °C to constant mass (*m*_f_), and the WS (%) was subsequently calculated using eq 2 given below.

2 $$WS\;\left(\%\right)=\frac{m_{i}-m_{f}}{m_{i}}\times 100$$

#### 2.2.8 WVP

The WVP of the films was determined gravimetrically in accordance with ASTM E96 after preconditioning at 50% equilibrium relative humidity, using glass permeability cups with a height of 3 cm and an internal diameter of 5 cm.^27^ The mass of the films was measured after being placed over silica gel (0% relative humidity) in a glass permeability cup sealed with silicone vacuum grease and an O-ring, and the bottles were subsequently stored in a water-vapor-saturated desiccator at 30 °C for 10 h. The mass of the sealed weighing bottle was recorded hourly, and the WVP of the films was calculated using eq 3.

3 $$WVP=\frac{Weight\;gain\;of\;the\;test\;cups\;\left(g\right)\times thickness}{film\;area\times time\times vapor\;pressure\;difference}\times 100$$

#### 2.2.9 Oxygen Permeability (OP)

The OP values of CH/ZnO^2%^ and CH/ZnO^2%^/CO-based films were determined with minor modifications based on the procedures reported by Rui et al.^28^ and Shen et al.^29^ For this purpose, the OP test was conducted by sealing a weighing bottle containing 3 g of Deoxidizer powder (reduced iron powder, sodium chloride, and activated carbon in a 0.5:1.5:1 ratio) with CH/ZnO^2%^ and CH/ZnO^2%^/CO-based films, in which oxygen consumption by iron powder and activated carbon generated a micro-oxygen environment that induced a pressure gradient driving external oxygen through the film. Bottles sealed with films were conditioned at an average temperature of 25 °C and an average relative humidity of 90%, weighed after an average exposure time of 7 days, and their mass changes were recorded at 24-h intervals. OP (g/s m^2^) was calculated using eq 4, and each type of film was tested in triplicate.

4 $$OP=\frac{weight\;of\;the\;flask\;containing\;deoxidizer\;powder\;\left(g\right)-cons\tan t\;weight\;of\;the\;flask\;\left(g\right)}{equilibrium\;time\;\left(s\right)\times test\;area\;of\;the\;film\;\left(m^{2}\right)}$$

#### 2.2.10 Color Evaluation

The L*, a*, and b* values of the films were measured with a Hunter colorimeter (Hunter Associates Laboratory, USA), where L*, a*, and b* correspond to lightness, the red–green axis, and the yellow–blue axis, respectively, and the overall color difference (ΔE*) was calculated using eq 5.

5 $$\Delta E=\sqrt{{\left(L^{*}-L_{0}^{*}\right)}^{2}+{\left(a^{*}-a_{0}^{*}\right)}^{2}+\left(b^{*}-b_{0}^{*}\right)}$$

#### 2.2.11 Light Transmittance and Transparency

The UV–visible transmittance of the films (15 mm × 40 mm) was recorded over the 200–800 nm range using a spectrophotometer (Agilent Technologies, USA, Cary 60 UV–vis) with each measurement performed in triplicate, and a blank cuvette served as the control, while the transparency of the films was calculated at 600 nm using eq 6.

6 $$Transparency\;\left(A/mm\right)=-{\log T}_{600}/thickness$$

#### 2.2.12 FTIR

The FTIR spectra of the films were recorded using a PerkinElmer Spectrum 100 FTIR system with a universal ATR sampling accessory, and data were collected over the 4000–500 cm^–1^ range.

#### 2.2.13 SEM

The surface and cross-sectional structures of the films were examined using SEM (Thermo Scientific Apreo S) after the cross sections were cryogenically fractured with liquid nitrogen, followed by critical point drying for moisture removal and sputter-coating with a 10 nm gold layer.

#### 2.2.14 DSC

Differential scanning calorimetry of the films was performed using a TA Instruments DSC Q20 (New Castle, UK) under a nitrogen purge of 30 mL min^–1^, with 7 mg samples encapsulated in sealed aluminum pans subjected to a 25–225 °C heating–cooling cycle at 10 °C min^–1^, employing a blank aluminum pan as the reference.

#### 2.2.15 Antioxidant Activity

The antioxidant activity of CH/ZnO^2%^ (control) and CH/ZnO^2%^/CO-based films was evaluated using DPPH and ABTS radical-scavenging assays, following a modified protocol based on a previous study.^30^ After immersion of 30 mg film samples in 5.0 mL deionized water for 24 h with shaking at 150 rpm and 25 °C overnight, 0.1 mL of the resulting film solution was mixed with 3.9 mL of DPPH solution prepared in 99.9% methanol, thoroughly shaken, and incubated in the dark at room temperature for 45 min. The absorbance of the supernatant was recorded at 517 nm using a UV–vis spectrophotometer (Agilent, Cary 60) with a 1 cm path length cuvette, using methanol as the blank, and the radical-scavenging activity (RSA, %) of the film samples was calculated accordingly eq 7, with all measurements performed in triplicate

7 $$DPPH\;RSA\;\left(\%\right)=\frac{A_{blank}\pm A_{sample}}{A_{blank}}$$

where *A*_blank_ is the absorbance of the control (DPPH solution without film) and *A*_sample_ is the absorbance of the test sample.

The ABTS cation radical was generated by mixing 7 mM ABTS with 2.45 mM potassium persulfate (2:1) and incubating the mixture in the dark at 25 °C for 16 h, after which the solution was diluted 40–60-fold with absolute ethanol, mixed thoroughly, and allowed to equilibrate for 5 min. After mixing 50 mg of film sample with approximately 5.0 mL of diluted ABTS aqueous solution (initial absorbance 0.70 ± 0.03 at 734 nm) and incubating under shaking in the dark at 37 °C for 30 min, the absorbance was measured at 734 nm using a UV–vis spectrophotometer (Agilent, Cary 60), and ABTS RSA (%) was calculated in triplicate using eq 8

8 $$ABTS\;RSA\;\left(\%\right)=\frac{A_{blank}\pm A_{sample}}{A_{blank}}$$

where *A*_blank_ is the absorbance value of the diluted ABTS aqueous solution and *A*_sample_ is the absorbance value of the ABTS aqueous solution in the presence of the sample.

#### 2.2.16 Statistical Analysis

All experiments were performed in triplicate, and the data are reported as mean ± standard deviation. The statistical analysis of the mean ± standard deviation obtained from at least three replicates was performed using ANOVA in SPSS software (professional version, v. 25.0), with significance considered at *p* < 0.05.

## 3 Results and Discussion

### 3.1 Electrophoretic Analysis of CH

As shown in Figure 1, a protein marker (PM) was used to estimate the molecular weights of the protein bands of CH obtained from leather industry trimming wastes. The electrophoretic patterns indicate that the CH extracted from leather industry trimming wastes possesses two peptide chains, α_2_ and α_1_, with molecular weights of 102 kDa and 114 kDa, respectively, and a β peptide chain of 172 kDa, as shown in the CH lane in Figure 1. Additionally, the obtained CH exhibits the characteristic bands of type I collagen molecules, including the α_1_ chain, α_2_ chain, and β chain.

**Figure 1:**
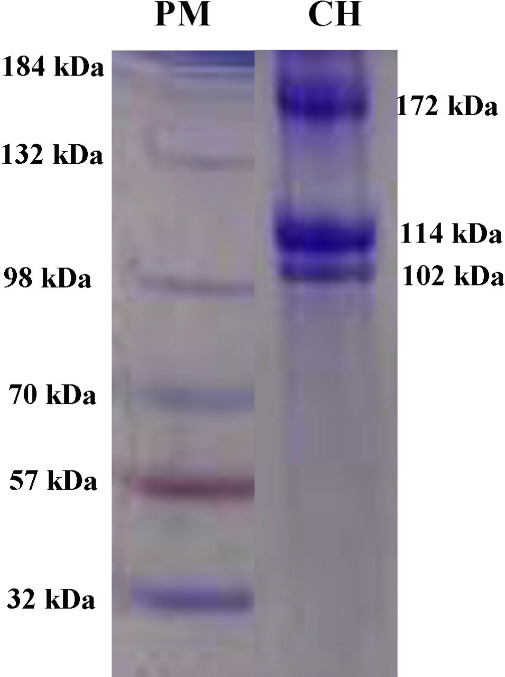
SDS-PAGE profile of the CH.

SDS-PAGE analyses of collagen and CHs have demonstrated distinct structural characteristics and enzymatic susceptibility: Rathod et al.^31^ reported that collagen exhibited two α bands (≈95–116 kDa) and a β band (≈220 kDa), reflecting the presence of unfolded polypeptide chains while confirming the native triple-helical structure, whereas Ahmad et al.^32^ observed that bromelain (30 and 50 U/g) effectively degraded buffalo hide collagen into smaller chains of approximately 62 kDa and 29 kDa, with additional fragments below 14 kDa appearing in the incubated samples.

### 3.2 Characterization of CH/ZnO-Based Films

#### 3.2.1 Thickness

Film thickness, governed by the polymer matrix, its components, and processing conditions, is a key parameter influencing the mechanical, optical, light transmittance, opacity, and WVP properties of biodegradable packaging materials and plays a decisive role in film selection for packaging applications.^33–35^ The thickness values of the CH and CH/ZnO-based films are summarized in Table 1, showing a slight but statistically significant increase (*p* < 0.05) with increasing ZnO content, from 0.071 mm for the control CH film to 0.100 mm for the CH/ZnO^4%^ film.

**Table 1:** Thickness, WS (%), MC (%) and WVP Values of CH and CH/ZnO-Based Films^a^

**Film samples**	**Thickness (mm)**	**WS (%)**	**MC (%)**	**WVP** **(×10^–11^ g/ms Pa)**
CH (control)	0.071 ± 0.008^c^	82.930 ± 1.947^a^	20.088 ± 0.382^a^	7.409 ± 0.259^a^
CH/ZnO^0.5%^	0.078 ± 0.006^c^	72.872 ± 1.052^b^	19.009 ± 0.317^b^	6.476 ± 0.204^b^
CH/ZnO^1%^	0.090 ± 0.007^b^	61.565 ± 1.054^c^	18.291 ± 0.415^c^	5.646 ± 0.208^c^
CH/ZnO^2%^	0.094 ± 0.004^b^	50.950 ± 0.777^e^	16.941 ± 0.422^d^	4.435 ± 0.274^e^
CH/ZnO^4%^	0.100 ± 0.004^a^	57.616 ± 0.955^d^	16.714 ± 0.440^d^	5.090 ± 0.198^d^

a Different letters (a–e) in the same column denote significant differences among sample groups as determined by Duncan’s test (*p* < 0.05).

The differing observations of Mousazadeh et al.^34^ and Roy et al.^36^ highlight the role of polymer matrix composition in determining film thickness. Mousazadeh et al.^34^ observed a notable increase in thickness following the incorporation of ZnO nanoparticles into modified gelatin films, attributed to elevated solid content, whereas Roy et al.^36^ found no significant change in gelatin/cellulose nanofiber films, suggesting that nanofibers promote a more compact network. These results suggest that nanoparticle-induced changes in film thickness are influenced by the characteristics of the matrix and the interactions between nanoparticles and the polymer.

#### 3.2.2 WS

Low WS is a critical requirement for packaging films, as it enhances water resistance and product integrity, ensuring stability under high humidity conditions and direct water contact.^37–39^ The WS (%) values of the CH and CH/ZnO-based films are presented in Table 1, with the control CH film exhibiting the highest WS (82.93%), while the CH/ZnO^2%^ film showed the lowest value (50.95%), representing an approximate reduction of 38.5%. A significant decrease (*p* < 0.05) in the WS (%) of CH/ZnO-based films was observed with increasing ZnO concentration from 0.5% to 2%, whereas a significant increase (*p* < 0.05) in WS (%) occurred when the ZnO concentration was further increased to 4% (Table 1). The effect of ZnO nanoparticles on WS (%) is highly dependent on polymer matrix architecture and nanoparticle loading, as demonstrated by Mousazadeh et al.^34^ for modified gelatin-based pH-sensitive films, which exhibited a nonlinear solubility behavior associated with polymer network disruption at higher ZnO levels, and by Roy et al.^36^ for gelatin/cellulose nanofiber-based nanocomposite films, which showed a consistent decrease in solubility attributed to nanofiber-induced network stabilization and limited water penetration.

#### 3.2.3 MC (%)

MC (%) reflects the water absorption capacity of film components and is a critical parameter influencing mechanical properties, shelf life, and microbial stability of films.^16,40,41^ The MC (%) of the CH and CH/ZnO-based films is presented in Table 1, showing a significant decrease (*p* < 0.05) from 20.088% in the control CH film to 16.714% in the CH/ZnO^4%^ film. The results reported by Roy et al.^36^ and Sasikala et al.^42^ consistently indicate that ZnO nanoparticle incorporation contributes to reduce MC (%) in biopolymer-based nanocomposite films, although the absolute values depend strongly on the polymer system. Roy et al. observed relatively low MCs (6.7–7.4%) in gelatin/cellulose nanofiber-based films, which can be attributed to the reinforcing and water-barrier effects of cellulose nanofibers that promote a dense polymer network and limit water uptake.^36^ Similarly, Sasikala et al. demonstrated that ZnO addition to gelatin/starch-based films decreased MC (%) by increasing structural compactness, reducing microstructural voids, and enhancing hydrophobicity, while the higher MC (%) of the control films was linked to the availability of free OH and NH_2_ groups capable of hydrogen bonding with water.^42^ Collectively, these findings suggest that ZnO nanoparticles, in combination with matrix-specific interactions and secondary biopolymers, play a critical role in modulating moisture affinity and barrier performance of bionanocomposite films.

#### 3.2.4 WVP

Low WVP is essential for food packaging films, as moisture transfer through hydrophilic domains affects shelf life and can be reduced by increasing hydrophobicity, while high WVP is associated with microbial growth, textural changes, and undesirable chemical and enzymatic reactions.^34,43,44^ The WVP of CH and CH/ZnO-based films significantly (*p* < 0.05) decreased from 7.409 × 10^–11^ g·m/(s·Pa) in the control CH film to 4.435 × 10^–11^ g·m/(s·Pa) in the CH/ZnO^2%^ film (≈40.2% reduction), then increased in the CH/ZnO^4%^ film (Table 1). The primary role of packaging is to limit moisture exchange, making low WVP desirable, and in ZnO-containing films, nanoparticles create complex channels that hinder water diffusion, enhancing water resistance and demonstrating their suitability for food applications. Similar to our findings, Roy et al.^36^ reported WVP values of (0.86–0.94) × 10^–9^ g·m/(m^2^·Pa·s) for ZnO-incorporated gelatin/cellulose nanofiber nanocomposite films, while Ubaid and Saini^45^ observed WVP values ranging from 10.21 to 28.93 × 10^–12^ g·m/(s·Pa) for grape seed protein hydrolysate films integrated with ZnO. Considering the obtained data, the CH/ZnO^2%^ formulation was chosen based on MC (%), WS (%), and WVP values, and the impact of varying concentrations of CO essential oil on the films’ structural, termal, mechanical, antioxidant and barrier characteristics was subsequently examined.

### 3.3 Characterization of CH/ZnO/CO-Based Films

#### 3.3.1 Thickness

Film thickness is a crucial factor affecting mechanical properties, including TS and EAB (%), as well as permeability and the visual appearance of packaged products.^46,47^ The thickness of CH/ZnO^2%^-based films increased significantly (*p* < 0.05) with CO addition, rising from 0.094 mm in the control CH/ZnO^2%^ film to 0.147 mm in the CH/ZnO^2%^/CO^8%^ film, corresponding to an approximate 56.4% increase (Table 2).

**Table 2:** Thickness, WS (%), MC (%) and WVP Values of CH/ZnO^2%^ and CH/ZnO^2%^/CO-Based Films^a^

**Film samples**	**Thickness (mm)**	**WS (%)**	**MC (%)**	**WVP** **(10^–11^ g/ms Pa)**	**OP** **(×10^–8^ g/m^2^ s)**
CH/ZnO^2%^ (control)	0.094 ± 0.004^e^	50.950 ± 0.777^a^	16.941 ± 0.422^a^	4.435 ± 0.274^a^	5.581 ± 0.457^a^
CH/ZnO^2%^/CO^2%^	0.105 ± 0.004^d^	45.680 ± 0.710^b^	15.805 ± 0.354^b^	3.967 ± 0.208^b^	4.624 ± 0.648^b^
CH/ZnO^2%^/CO^4%^	0.124 ± 0.002^c^	41.497 ± 1.397^c^	14.475 ± 0.210^c^	3.562 ± 0.253^c^	4.048 ± 0.586^c^
CH/ZnO^2%^/CO^6%^	0.134 ± 0.008^b^	37.117 ± 1.615^d^	13.135 ± 0.585^d^	2.997 ± 0.127^d^	3.484 ± 0.367^d^
CH/ZnO^2%^/CO^8%^	0.147 ± 0.004^a^	35.775 ± 0.860^e^	12.362 ± 0.317^e^	2.435 ± 0.130^e^	2.501 ± 0.504^e^

a Different letters (a–e) in the same column denote significant differences among sample groups as determined by Duncan’s test (*p* < 0.05).

Thicker films generally enhance barrier properties and water resistance, while thin films reduce TS, EAB (%), and WVP, and overly thick films may affect appearance; these effects are attributed to interactions between essential oils and the polymer matrix, where hydrophobic oil droplets disrupt polymer network, causing structural rearrangements and a bulkier microstructure.^46,48,49^ Both Rathod et al.^31^ and Huang et al.^50^ demonstrated that incorporation of *Calendula* extract into biopolymer-based films increases film thickness, with Rathod et al.^31^ reporting thicknesses of 0.01–1.1 mm in collagen-based films and Huang et al.^50^ observing 0.058–0.226 mm in carboxymethyl cellulose/poly­(vinyl alcohol) matrixes, highlighting the concentration-dependent effect of CO on film morphology.

#### 3.3.2 WS (%)

WS (%) of biodegradable films is a key indicator for assessing their hydrolytic resistance and structural stability under aqueous conditions.^51,52^ The WS (%) of CH/ZnO^2%^-based films decreased significantly (*p* < 0.05) with increasing CO concentration, dropping from 50.950% in the control CH/ZnO^2%^ film to 35.775% in the CH/ZnO^2%^/CO^8%^ film, corresponding to an approximate 29.8% reduction (Table 2). Films with lower WS (%) are preferred for packaging, and in CH/ZnO^2%^-based films, higher CO content reduces WS (%) by introducing hydrophobic essential oil compounds that form hydrogen-bond interactions with the polymer, limiting CH–water interactions and producing films more resistant to moisture.^53,54^ Incorporation of essential oils into gelatin-based films reduces WS (%) by enhancing hydrophobicity and promoting molecular interactions, including cross-linking and interactions between film hydroxyl groups and oil constituents, as shown for lemon oil^52^ and mandarin oil in gelatin/pectin films.^51^

#### 3.3.3 MC (%)

MC (%) critically influences the protective performance and physicochemical properties of films, as water molecules act as plasticizers that affect moisture management and overall product quality.^52,55,56^ The MC (%) of CH/ZnO^2%^-based films decreased significantly (*p* < 0.05) with increasing CO content, dropping from 16.941% in the control film to 12.362% in the CH/ZnO^2%^/CO^8%^ film, corresponding to a 4.579% reduction (Table 2). Films intended for packaging should have low MC (%) to ensure stability and water resistance, as interactions between the polymer matrix and EO components can modify water-binding capacity, with higher MC (%) linked to the hydrophilic nature and dense hydrogen bonding of the films.^46,48,57^ Fathimoghadam et al.^52^ reported that lemon essential oil incorporation into gelatin films reduces MC (%) by promoting covalent interactions with polymer chains, limiting hydroxyl and amino group availability and weakening polymer–water hydrogen bonding, while Jafari et al.^58^ showed that increasing anise oil in gelatin/alginate films lowers MC (%) by loosening the microstructure, increasing free volume, and reducing polymer–water interactions.

#### 3.3.4 WVP

WVP, a critical physicochemical parameter of packaging materials, determines the degree to which films restrict moisture migration from the product to the ambient environment, reflecting their barrier performance under high-humidity conditions.^51,59,60^ Incorporation of CO into CH/ZnO^2%^-based films reduced WVP in a concentration-dependent manner, decreasing from 4.435 × 10^–11^ g/m·s·Pa in the control to 2.435 × 10^–11^ g/m·s·Pa in the CH/ZnO^2%^/CO^8%^ film (≈45.1% reduction), with significant (*p* < 0.05) improvements in barrier properties attributed to modifications in film microstructure and polymer–water interactions (Table 2). Although biopolymer-based films typically exhibit higher WVP due to their hydrophilic nature, incorporation of hydrophobic CO with Tween 80 reduces WVP by creating low-affinity regions for water diffusion, and increasing CO concentration further enhances barrier properties through hydrophobicity and improved molecular integration.^61,62^ Similarly, Fathiraja et al.^60^ reported that fish scale gelatin/agar/chitosan and composite films incorporating clove essential oil exhibited WVP values of 0.64 and 0.25 × 10^–10^ g/m·s·Pa, respectively, and emphasized that WVP is influenced by polymer chain mobility, inter- and intramolecular interactions, film integrity, and the hydrophilic/hydrophobic balance. Despite the marked improvement in water barrier performance of the CH/ZnO^2%^/CO^8%^ films (2.435 × 10^–11^ g/m·s·Pa), their WVP remains higher than that of commercial LDPE films (∼0.0070 × 10^–11^ g/m·s·Pa) and is substantially lower than the WVP reported for xylan/poly­(vinyl alcohol) films (10.45 × 10^–11^ g/m·s·Pa), highlighting an intermediate barrier performance relative to synthetic and other biopolymer-based films.^63,64^

#### 3.3.5 OP

OP is a critical property of packaging films, as oxygen penetration governs oxidation and influences product quality and oxidative stability, while films with high oxygen barrier capacity effectively prevent oxidative deterioration of packaged products.^56,65^ The OP of CH/ZnO^2%^-based films decreased significantly (*p* < 0.05) with increasing CO concentration, dropping from 5.581 × 10^–8^ g/m^2^·s in the control CH/ZnO^2%^ film to 2.501 × 10^–8^ g/m^2^·s in the CH/ZnO^2%^/CO^8%^ film, corresponding to an approximate 55.2% reduction (Table 2), with CH/ZnO^2%^/CO^8%^ films showing the lowest OP and the greatest potential to inhibit oxygen transmission in practical applications. Previous studies have shown that incorporating bioactive compounds significantly reduces OP, with Shah et al.^66^ reporting a decrease from 6.16 to 3.58 × 10^–7^ g/m^2^·s in carboxymethyl cellulose/sodium alginate films after adding turmeric essential oil nanoemulsion and gallic acid, Guo et al.^67^ observing a reduction from 6.32 to 5.86 × 10^–10^ g/m^2^·s following thyme essential oil addition, and Rui et al.^28^ reporting OP of chitosan films with clove essential oil nanoemulsion ranging from 23.62 to 34.41 × 10^–4^ g/m^2^·s.

#### 3.3.6 Mechanical and Antioxidant Properties

TS, EAB (%), and EM are critical mechanical parameters defining the strength and flexibility of films, with both TS and EAB (%) being particularly important for maintaining structural integrity during packaging, transport, and storage.^59,68^ The TS of CH/ZnO^2%^-based films significantly decreased (*p* < 0.05) with increasing CO content, dropping from 35.610 MPa in the CO-free control to 19.707 MPa in the CH/ZnO^2%^/CO^8%^ film, corresponding to an approximate 80.7% reduction (Table 3).

**Table 3:** TS, EAB (%) and EM of CH/ZnO^2%^ and CH/ZnO^2%^/CO-Based Films^a^

**Film samples**	**TS (MPa)**	**EAB (%)**	**EM (MPa)**	**DPPH (%)**	**ABTS (%)**
CH/ZnO^2%^ (control)	35.610 ± 1.793^a^	51.190 ± 1.435^e^	71.369 ± 3.732^a^	14.404 ± 0.915^e^	13.286 ± 0.239^e^
CH/ZnO^2%^/CO^2%^	30.885 ± 1.358^b^	56.800 ± 0.930^d^	59.759 ± 4.081^b^	23.015 ± 1.296^d^	21.044 ± 0.256^d^
CH/ZnO^2%^/CO^4%^	27.277 ± 1.083^c^	65.237 ± 1.702^c^	51.325 ± 2.628^c^	35.357 ± 1.437^c^	31.344 ± 0.421^c^
CH/ZnO^2%^/CO^6%^	23.583 ± 1.251^d^	68.905 ± 2.190^b^	45.463 ± 2.516^d^	48.231 ± 1.275^b^	40.621 ± 0.335^b^
CH/ZnO^2%^/CO^8%^	19.707 ± 1.078^e^	83.535 ± 2.115^a^	37.260 ± 0.775^e^	60.735 ± 0.881^a^	57.635 ± 0.218^a^

a Different letters (a–e) in the same column denote significant differences among sample groups as determined by Duncan’s test (*p* < 0.05).

Although combining essential oils can enhance functionality, interactions between components may reduce film TS by disrupting polymer–polymer hydrogen bonding and creating hydrophobic domains; SEM analysis shows structural irregularities and hollow cavities weaken the matrix, so balancing essential oil concentration is crucial to optimize mechanical performance while preserving antioxidant properties for packaging.^49,69,70^ These findings align with previous reports, where Huang et al.^50^ observed a TS decrease from 42.8 to 13.2 MPa in carboxymethyl cellulose/poly­(vinyl alcohol) films with CO addition up to 5%, and Shah et al.^71^ reported a reduction from 18.46 to 7.33 MPa in gelatin/kappa-carrageenan films upon lemon essential oil incorporation due to oil–matrix interactions disrupting the polymer network. LDPE and HDPE are among the most widely used packaging materials; LDPE has been reported to exhibit TS values of up to 15.10 MPa^72^ and 18.96 MPa,^73^ whereas HDPE shows consistently higher TS values (26–29 MPa) than LDPE (12–14 MPa), attributable to its greater molecular orientation and higher crystalline density.^74^

The EAB (%) of CH/ZnO_2%_-based films increased significantly (*p* < 0.05) with rising CO content, from 51.190% in the CO-free control to 83.535% in the CH/ZnO^2%^/CO^8%^ film, corresponding to an approximate 63.1% increase (Table 3). The EAB (%) enhancement in CO-containing films is attributed to the plasticizing effect of CO, which weakens intermolecular bonds, increases molecular mobility, and facilitates stress distribution, resulting in a more flexible polymeric network.^48,49,75^ Incorporation of essential oils into biopolymer films enhanced EAB, with EAB increasing from 19.21% to 25.41% in fish scale gelatin/agar/chitosan films containing clove oil^60^ and from 31.43% to 56.60% in gelatin/kappa-carrageenan films with 0.1% lemon oil.^71^

The EM of CH/ZnO^2%^-based films decreased significantly (*p* < 0.05) with increasing CO content, from 71.369 MPa in the CO-free control to 37.260 MPa in the CH/ZnO2%/CO8% film, corresponding to an approximate 49.0% reduction (Table 3). The EM decreased with CO addition as its plasticizing effect and hydrophobic interactions with the polymer backbone weakened chain cohesion, while embedded CO droplets created discontinuities in the matrix, making films deform more easily under tension and reducing stiffness, as confirmed by SEM analysis.^23,76,77^ Rashidi et al.^23^ observed a significant decrease in EM with increasing mint essential oil in opopanax gum/gelatin films, while Babu et al.^77^ reported that *Ocimum tenuiflorum* essential oil reduced the EM of psyllium husk–methylcellulose films, indicating lower film stiffness. The EM values of CH/ZnO^2%^-based films are comparable to those of conventional food-packaging polymers, with LDPE exhibiting an EM of 460 MPa^72^ and HDPE showing an EM of 435.1 MPa.^78^

The RSA of the films, evaluated by DPPH and ABTS assays, is enhanced by the incorporation of ZnO nanoparticles and active biocomposite additives, as nanoparticle internalization and high surface area improve antioxidant performance in packaging systems.^79,80^ The DPPH assay, widely used to evaluate antioxidant radical-scavenging ability, is based on hydrogen or electron donation, whereby the dark-purple stabilized DPPH radical is reduced to a light-yellow form with a concomitant decrease in absorbance at 517 nm, reflecting the termination of oxidative chain reactions by antioxidant microconstituents.^79,81,82^ The DPPH and ABTS RSA values of the produced CH/ZnO^2%^ and CH/ZnO^2%^/CO-based films containing different CO concentrations are presented in Table 3. In CH/ZnO^2%^-based control films, the moderate antioxidant activity observed in both DPPH (14.404%) and ABTS (13.286%) assays is attributed to electron donation from ZnO and peptide-derived amino acids in the CH matrix, while being limited by reactive oxygen species generation from ZnO and depletion of electron-donating hydroxyl groups in CH during radical reactions.^83–86^ These findings indicate that the CH/ZnO^2%^ matrix exhibits inherently limited antioxidant activity, making it insufficient for effectively suppressing free-radical-induced oxidative processes.

The antioxidant activity of CH/ZnO^2%^/CO-based films increased significantly in a concentration-dependent manner (*p* < 0.05), attributable to the strong radical-scavenging capacity of CO-derived phenolic compounds (e.g., caffeic and chlorogenic acids, flavonoids, carotenoids, and polyphenols) and the diffusion of water-extractable antioxidant constituents from the film matrix during incubation.^85,87,88^ The CH/ZnO^2%^/CO^8%^ film exhibited the highest antioxidant activity, as the increased CO loading at 8% enhanced the reservoir of releasable antioxidants, resulting in markedly higher DPPH (60.735%) and ABTS (57.635%) scavenging efficiencies.^85^ Both DPPH and ABTS assays exhibited consistent enhancement trends, attributed to phenolic antioxidants in CO that neutralize free radicals via hydrogen atom or single-electron transfer mechanisms, with higher CO contents further increasing the concentration and release of antioxidant constituents from the film matrix.

#### 3.3.7 Color

Color is a critical factor influencing consumer acceptance of edible films, as it affects both appearance and functional properties.^56,89^ The L* values of CH/ZnO^2%^-based films decreased significantly (*p* < 0.05) with increasing CO content, from 83.648 in the CO-free control to 73.281 in the CH/ZnO^2%^/CO^8%^ film, corresponding to an approximate 12.5% reduction (Table 4). Consistent with our results, L* decreased from 90.5 to 77.0 in gelatin/chitosan films with 2% rosemary essential oil;^90^ it also declined in three-layer furcellaran/chitosan/gelatin hydrolysate films with increasing curcumin ethanol extract and lemongrass essential oil,^91^ and dropped significantly with higher orange essential oil content in gelatin/calcium caseinate films, indicating reduced film lightness.^89^

**Table 4:** Color Values of CH/ZnO^2%^ and CH/ZnO^2%^/CO-Based Films^a^

**Film samples**	L*	a*	b*	ΔE*
CH/ZnO^2%^ (control)	83.648 ± 0.574^a^	–1.581 ± 0.065^e^	4.733 ± 0.128^a^	4.807 ± 0.336^a^
CH/ZnO^2%^/CO^2%^	81.373 ± 0.533^b^	–1.200 ± 0.086^f^	7.768 ± 0.285^b^	8.341 ± 0.370^b^
CH/ZnO^2%^/CO^4%^	76.282 ± 0.525^c^	–1.058 ± 0.082^c^	19.987 ± 0.469^c^	20.972 ± 0.402^c^
CH/ZnO^2%^/CO^6%^	74.298 ± 0.294^d^	–0.798 ± 0.055^b^	21.751 ± 0.340^d^	22.774 ± 0.312^d^
CH/ZnO^2%^/CO^8%^	73.281 ± 0.378^e^	–0.065 ± 0.045^a^	25.142 ± 0.479^e^	26.950 ± 0.391^e^

a Different letters (a–e) in the same column denote significant differences among sample groups as determined by Duncan’s test (*p* < 0.05).

The a* values of CH/ZnO^2%^-based films increased significantly (*p* < 0.05) with rising CO content, from −1.581 in the CO-free control to −0.065 in the CH/ZnO2%/CO^8%^ film, corresponding to an approximate 95.9% increase (Table 4). Similar to our findings, a* increased from 0.9 to 2.9 in gelatin/chitosan films with 2% rosemary essential oil;^90^ a similar increase was observed in three-layer furcellaran/chitosan/gelatin hydrolysate films with curcumin ethanol extract and lemongrass essential oil,^91^ and values significantly rose with higher orange essential oil content in gelatin/calcium caseinate films.^89^

The b* values of CH/ZnO^2%^-based films increased significantly (*p* < 0.05) with rising CO content, from 7.768 at 2% CO to 25.142 at 8% CO (≈224% increase), while the CO-free control showed a b* value of 4.733 (Table 4). Consistent with the present findings, b* increased from 10.0 to 53.5 in gelatin/chitosan films with 2% rosemary essential oil;^90^ it was higher in furcellaran/chitosan/gelatin trilayer films with curcumin ethanol extract and lemongrass essential oil,^91^ and rose significantly with more bitter orange essential oil in gelatin/calcium caseinate films, showing that film color strongly depends on matrix composition and essential oil concentration.^89^

The ΔE* values of CH/ZnO^2%^-based films increased significantly (*p* < 0.05) with rising CO content, from 8.341 at 2% CO to 26.950 at 8% CO (≈223% increase), while the CO-free control showed a ΔE* value of 4.807 (Table 4). In line with our results, ΔE* increased from 10.1 to 54.3 in gelatin/chitosan films with 2% rosemary essential oil^90^ and rose significantly in gelatin/calcium caseinate films with higher bitter orange essential oil content, reflecting the effect of the EO’s phenolic composition and intrinsic yellow coloration.^89^

#### 3.3.8 Light Transmittance and Transparency

Efficient UV and visible light shielding is crucial to safeguard packaged products, with optical characteristics, including surface color, light transmittance, and clarity, indicating a film’s barrier performance and affecting its visual appeal, commercial value, and applicability across different uses.^59,90,92,93^ The UV–visible transmittance of the control CH/ZnO^2%^ and CH/ZnO^2%^/CO films over the 200–800 nm wavelength range is presented in Table 5. Compared to the control film, CO-incorporated films exhibited markedly lower UV transmittance at 280 nm, and their visible light transmittance decreased progressively across all wavelengths with increasing essential oil concentration (Table 5). The obtained findings demonstrate that CO essential oil effectively reduced UV and visible light passage through CH/ZnO^2%^-based films.

**Table 5:** Light Transmittance and Transparency Values of CH/ZnO^2%^ and CH/ZnO^2%^/CO-Based Films^a^

	**Light Transmittance (%) at Different Wavelengths (nm)**								
**Film samples**	**200**	**280**	**350**	**400**	**500**	**600**	**700**	**800**	**Şeffaflık**
CH/ZnO^%2^ (control)	0.000	28.331 ± 0.302	57.677 ± 0.147	67.557 ± 0.071	71.319 ± 0.161	75.051 ± 0.298	84.403 ± 0.205	86.601 ± 0.098	1.791 ± 0.071^e^
CH/ZnO^%2^/CO^%2^	0.000	12.147 ± 0.965	40.155 ± 0.872	47.342 ± 0.474	52.085 ± 1.724	61.523 ± 1.773	70.391 ± 1.817	78.538 ± 0.117	2.232 ± 0.081^d^
CH/ZnO^%2^/CO^%4^	0.000	8.464 ± 0.786	34.679 ± 1.489	39.109 ± 1.016	49.105 ± 1.433	55.458 ± 1.426	62.062 ± 2.621	69.523 ± 0.107	2.509 ± 0.153^c^
CH/ZnO^%2^/CO^%6^	0.000	5.157 ± 0.671	31.545 ± 1.849	34.935 ± 1.082	46.554 ± 1.927	50.569 ± 2.031	54.211 ± 2.103	65.134 ± 0.137	2.809 ± 0.095^b^
CH/ZnO^%2^/CO^%8^	0.000	4.047 ± 0.519	25.373 ± 0.529	29.323 ± 1.045	37.332 ± 0.940	41.935 ± 0.921	48.983 ± 0.862	59.935 ± 0.063	3.171 ± 0.145^a^

a Different letters (a–e) in the same column denote significant differences among sample groups as determined by Duncan’s test (*p* < 0.05).

Similar to our findings, light transmittance was markedly reduced in fish skin gelatin-based films with bergamot and lemongrass essential oils, showing very low UV (200–280 nm) and decreased visible-light transmittance (44.58–56.48%) depending on oil type and concentration;^92^ strong UV barrier properties were also observed in fish protein isolate/gelatin films, further enhanced by basil leaf essential oil due to light scattering from lipid droplets.^94^ Clove essential oil in gelatin/myofibrillar films sharply decreased transmittance at 600 nm to 35.75%, attributed to UV-absorbing phenolics, aromatic amino acids, and pigments,^95^ while lemongrass oil in fish gelatin/pectin composites similarly reduced transmittance across 350–800 nm, especially at higher concentrations, due to interactions with the polymer matrix.^93^

Transparent films are generally preferred in packaging systems due to their greater applicability and consumer acceptability, whereas higher measured transparency values can actually indicate lower perceived film transparency.^92,94^ The transparency of CH/ZnO^2%^-based films increased significantly (*p* < 0.05) with rising CO content, from 1.791 in the CO-free control to 3.171 in films with 8% CO, representing an approximate 77% increase (Table 5). The reduction in transparency with increasing CO content can be attributed to the natural yellow pigmentation of the oil, and to increased light scattering within the emulsion matrix. Transparency decreased in protein- and polysaccharide-based films with essential oil incorporation: basil leaf oil reduced it in fish protein isolate/gelatin films due to coloring compounds and heterogeneous networks;^94^ clove oil lowered it in gelatin/myofibrillar films up to 6.37 at 9% concentration;^95^ lemongrass oil droplets scattered light in fish gelatin/pectin films;^93^ mint oil decreased it in opopanax gum/gelatin films;^23^ and higher orange oil in gelatin/calcium caseinate films significantly diminished transparency, showing that elevated essential oil content generally reduces light transmission regardless of matrix type.^89^

#### 3.3.9 FTIR

FTIR analysis was conducted to investigate the chemical structure of CH/ZnO^2%^ films and CH/ZnO^2%^/CO films with varying CO concentrations, with spectra recorded over the 500–4000 cm^–1^ range. As shown in Figure 2, the FTIR spectra exhibit distinct peaks corresponding to the functional groups in the films, and the observed shifts in characteristic absorption bands, together with changes in peak intensity and profiles, indicate intermolecular interactions primarily involving the hydroxyl groups of ZnO and CO and the amino groups of CH, providing insight into the compatibility of the film components.

**Figure 2:**
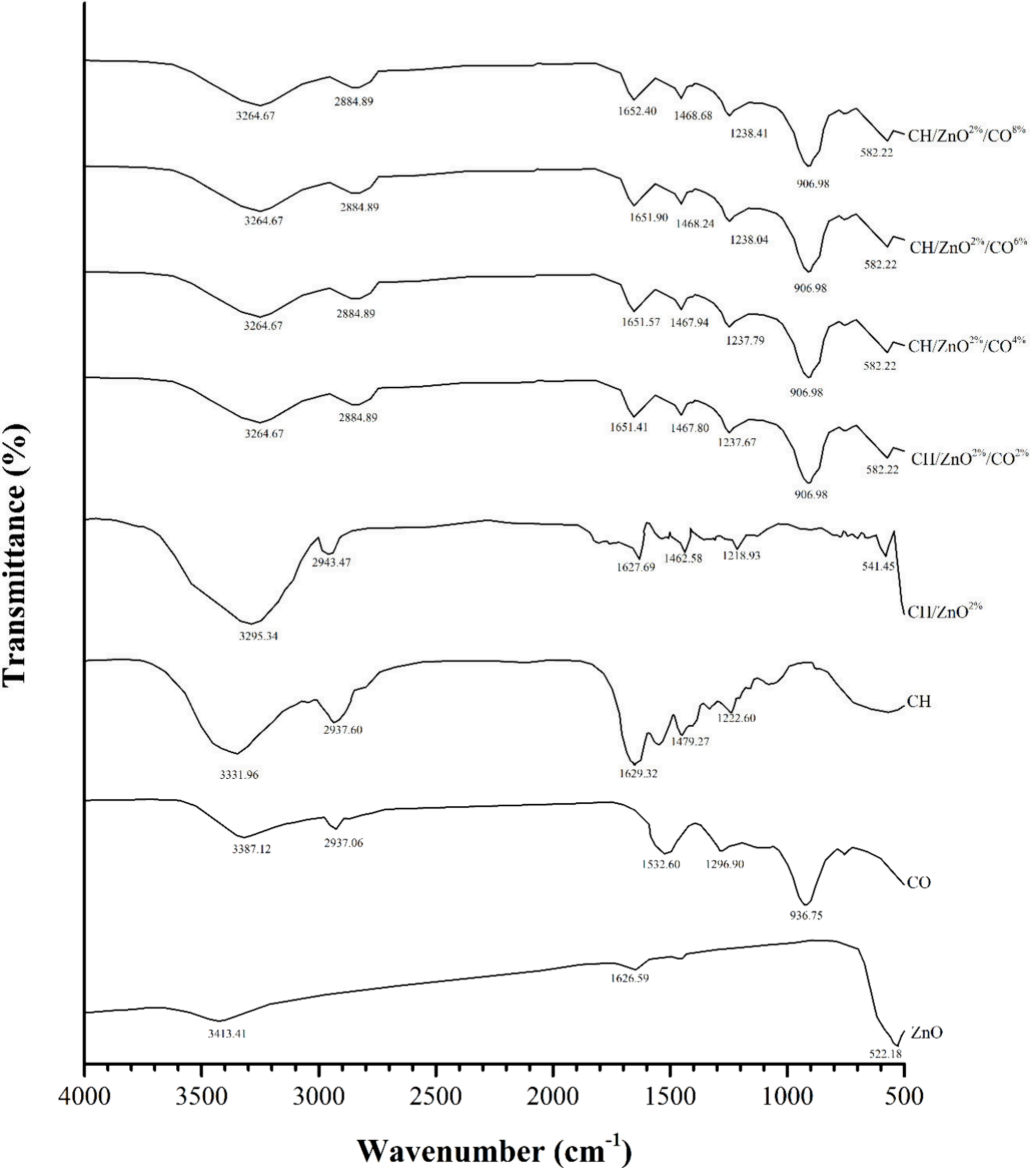
FTIR spectra of CH/ZnO^2%^ and CH/ZnO^2%^/CO films incorporated with CO.

Absorption bands around 3413.41 cm^–1^ were attributed to O–H stretching from hydroxyl groups, likely arising from adsorbed moisture or surface hydroxylation of ZnO by CH molecules, while the same band in ZnO-added films was also associated with N–H stretching coupled with hydrogen-bonded O–H stretching, suggesting the formation of CH–ZnO hydrogen bonding, consistent with similar bands reported at 3410 cm^–1^ by Abasalta et al.^96^ and 3450 cm^–1^ by Farazin et al.^97^ Interatomic vibration bands below 1000 cm^–1^, characteristic of metal oxides, were observed for ZnO, including a bending vibration at 522.18 cm^–1^, consistent with Zn–O-related bands reported at 538.33 cm^–1^ by Balaure et al. (98), 650 cm^–1^ by Abasalta et al.,^96^ and 435 cm^–1^ by Farazin et al.^97^

With the incorporation of ZnO into CH films, the O–H/–NH stretching band shifted to lower wavenumbers and decreased in intensity, indicating enhanced hydrogen bonding and cross-linking that reduce free hydroxyl groups, promote a more compact polymer network, and improve TS, EM, and water barrier properties, consistent with the findings of Navaei et al.^99^ and Sachin et al.,^100^ as also observed in SEM analyses. Similarly, Janicijevic et al.^101^ reported multiple characteristic peptide/protein bands in gelatin films, and Ahmadi et al.^102^ observed that nanoparticle incorporation caused only slight changes in peak intensities. Minor shifts in the amide I (1629.32 to 1627.96 cm^–1^), amide II (1479.27 to 1462.58 cm^–1^), and amide III (1222.60 to 1218.93 cm^–1^) bands in CH/ZnO^2%^ films, together with hydroxyl-related spectral changes and reduced amide band intensity, indicate ZnO–polymer interactions involving CH carboxyl groups, confirming nanoparticle stabilization within a stable biocomposite matrix that underlies the enhanced mechanical, barrier, and functional properties, consistent with literature reports.^98,100,103^

The FTIR spectrum of CO essential oil exhibited multiple characteristic peaks: O–H stretching at 3387.12 cm^–1^, C–H stretching at 2937.06 cm^–1^, aromatic ring vibrations at 1532.60 cm^–1^, phenol or tertiary alcohol O–H bending at 1296.60 cm^–1^, and cyclohexane ring vibrations in methylene groups at 936.75 cm^–1^, consistent with Huang et al.,^50^ who identified similar functional groups in CO extract. The Amide B band attributed to asymmetric CH_2_ stretching in CH films at 2943.47 cm^–1^ shifted to a lower wavenumber (2884.89 cm^–1^) in CH/ZnO^2%^/CO-based films, indicating the incorporation of hydrophobic components.^101,104,105^ In CH/ZnO^2%^/CO-based films, the amide A (O–H/N–H stretching) band shifted from 3295.34 to 3264.67 cm^–1^ and decreased in intensity with CO incorporation, indicating enhanced hydrogen bonding, reduced −OH stretching, and fewer polar interactions among components, which correlate with lower WVP and WS (%) while maintaining overall structural characteristics.^105,106^ Furthermore, the shifts of the amide I (1627.69–1651.41 cm^–1^), amide II (1462.58–1467.80 cm^–1^), and amide III (1218.93–1237.67 cm^–1^) bands toward higher wavenumbers in CH/ZnO^2%^/CO-based films indicated minor conformational sensitivity without disruption of collagen triple-helix integrity, while reflecting weakened protein–protein interactions at higher CO levels, in agreement with the observed reductions in TS and EM and the morphological features revealed by SEM, and consistent with literature reports.^98,103,105^ A characteristic absorption at 936.75 cm^–1^, attributed to cyclohexane ring vibrations of methylene groups, was present in all CH/ZnO^2%^/CO-based films but absent in the control CH/ZnO^2%^ film, confirming the incorporation of CO-related compounds. Overall, the FTIR spectra of the composite films exhibited similar major sharp peaks with varying amplitudes, indicating that CO incorporation directly affected the molecular interactions of protein chains within the CH/ZnO^2%^/CO film matrix.

#### 3.3.10 SEM

The surface morphology of the films is largely determined by the intrinsic properties of their constituents, their interactions, and processing conditions;^107^ consequently, SEM was employed to evaluate how the component distribution within the matrix impacts the films’ properties. Figure 3 shows SEM micrographs of the surfaces and cross sections of CH/ZnO^2%^ and CH/ZnO^2%^/CO films, providing valuable insights into the microstructure of CO-containing CH/ZnO^2%^ films. The surface of the CH/ZnO^2%^ control film appeared uniform and even, free from defects such as cracks, holes, or pores, consistent with Bhatia et al.,^89^ who noted a homogeneous structure in the gelatin-based control sample.

**Figure 3:**
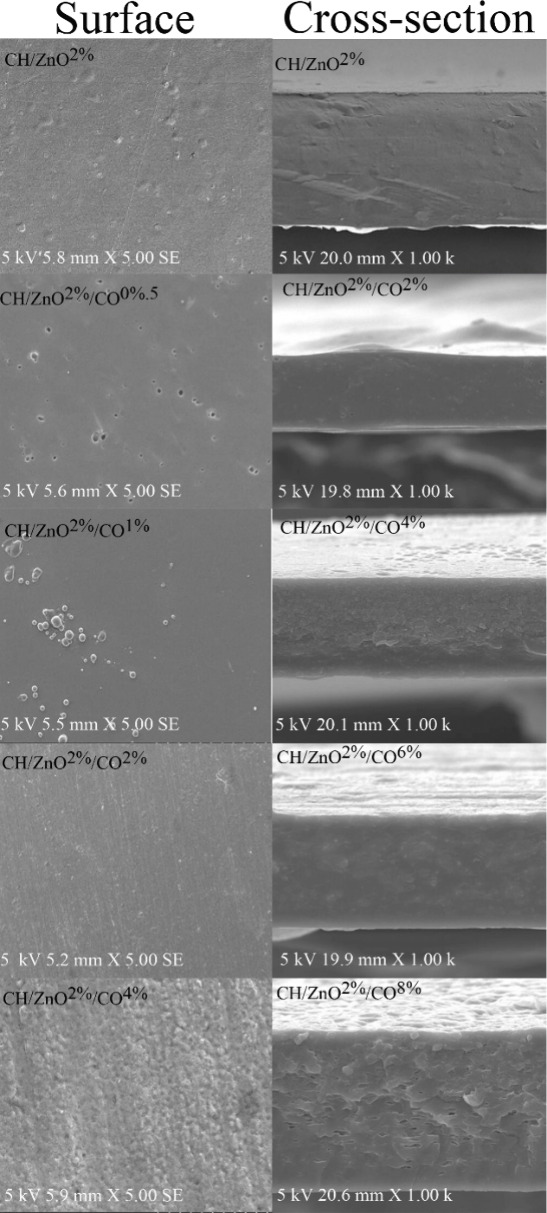
SEM images of CH/ZnO^2%^ and CH/ZnO^2%^/CO-based films.

Malihi et al.^108^ observed that incorporating additives, enzymes, or plant-derived oils enhanced surface roughness in fish gelatin-based films, aligning with the morphological irregularities and increased surface roughness seen in CH/ZnO^2%^ films containing CO. The addition of plant oils, owing to their hydrophobic characteristics, can create a heterogeneous matrix with an irregular microstructure,^108^ whereas control porcine and bovine gelatin films demonstrated better structural integrity than those containing peppermint oil.^107^

As observed in Figure 4, increasing concentrations of CO essential oil in CH/ZnO^2%^/CO films resulted in reduced surface smoothness and compactness, consistent with Huang et al.,^50^ who reported that carboxymethyl cellulose/poly­(vinyl alcohol) composite films with higher CO essential oil concentrations exhibited rough, discontinuous, and nonhomogeneous surfaces with increased porosity. This effect may result from limited interactions between the polymer and oil in the film matrix, potentially causing a decrease in the material’s mechanical integrity.^89,108^ Oil droplets can distribute within the protein matrix, creating a layered, noncontinuous arrangement that reduces interactions between protein molecules,^109^ and Huang et al.^50^ additionally identified complex and irregular architectures in the films’ cross-sectional structures via SEM. The films’ cross-sectional SEM images (Figure 4) demonstrate that the control film possesses a denser structure relative to the CO-containing samples. Based on the cross-sectional images, films containing CO essential oil exhibited certain structural cracks, consistent with observations by Bhatia et al.,^107^ who reported crack formation in films loaded with peppermint essential oil. The hydrophobic nature of essential oils can create microvoids during film drying as water migrates, contributing to the microcracks that compromise the mechanical performance of films containing CO essential oil.^108,110^ Malihi et al.^108^ observed that higher levels of *Oliveria decumbens* Vent. oil enhanced hydrophobic regions, which at 3% created continuous capillary-like structures leading to surface cracks, and that the cross-sectional micrographs of films containing the oil were rougher and less continuous than the denser control fish gelatin film.

**Figure 4:**
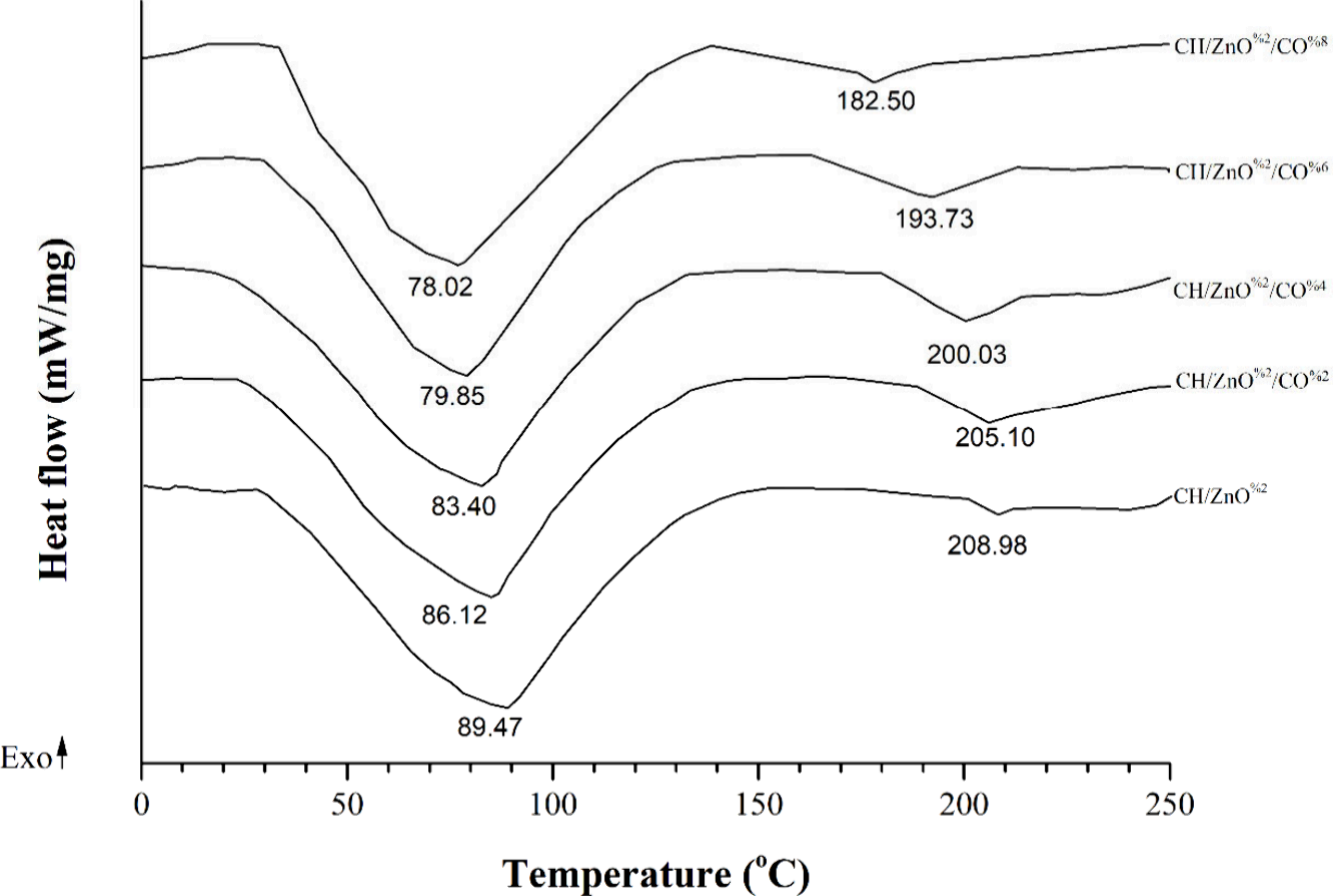
DSC thermograms of CH/ZnO^2%^ and CH/ZnO^2%^/CO-based films.

#### 3.3.11 Differential Scanning Calorimetry (DSC)

Villasante et al.^111^ reported that thermodynamic properties under varying conditions, such as temperature, gas pressure, or atmosphere, are useful for assessing film quality, with DSC peaks reflecting collagen’s thermal denaturation and fiber stability, while Ding et al.^112^ highlighted that thermal stability is a crucial property for the practical use of gelatin-based films. Figure 4 presents DSC curves of the CH/ZnO^2%^ and CH/ZnO^2%^/CO-based films.

Nunes et al.^113^ observed that the DSC thermograms of gelatin-based control films showed an endothermic peak at 104.50 °C, whereas the first endothermic peak of the CO-free control CH/ZnO^2%^ film, appearing at 89.47 °C, is associated with the evaporation of free water and melting of the oil.^113–115^ Mutlu^116^ observed that the control gelatin/guar gum-based film exhibited an endothermic peak at 61.44 °C, corresponding to the melting of gelatin’s triple-helix crystalline structure. Relative to the control, higher CO levels resulted in reduced endothermic temperatures; in agreement with Mei et al.,^114^ films incorporating Chinese fir oil showed lower temperatures, as the oil functioned as a softening agent influencing the films’ mechanical behavior. Following the addition of 2%, 4%, 6%, and 8% CO, the values decreased to 86.12 °C, 83.40 °C, 79.85 °C, and 78.02 °C, in agreement with Zong et al.,^117^ which reported a reduction in the endothermic temperature of gelatin/Polydextrose matrixes from 72.79 to 50.77 °C after incorporating camellia oil.

The second peak, occurring at approximately 250 °C, is associated with collagen degradation,^115^ while Nunes et al.^113^ reported that gelatin-based control films exhibited an endothermic peak at 225.03 °C. As indicated by the second endothermic peak, incorporating CO into the formulation affected the films’ heat resistance, with melting temperatures of 208.98 °C, 205.10 °C, 200.03 °C, 193.73 °C, and 182.50 °C recorded for films containing 0%, 2%, 4%, 6%, and 8% CO, respectively. Both Nunes et al.^113^ and Zong et al.^117^ observed that incorporation of green tea extract or camellia oil decreases the melting or endothermic peak temperatures in gelatin-based films by weakening intermolecular forces and disrupting the polymer network due to depolymerization or the oils’ hydrophobic properties.

## 4 Conclusion

In this study, adopting a circular economy approach, CH was initially obtained from leather industry trimming waste through enzymatic hydrolysis. In the second stage, aiming to enhance the water barrier performance of CH-based films, CH films were fabricated incorporating ZnO nanoparticles at different levels (0–4%) and assessed for thickness, MC (%), WS (%), and WVP. After determining that CH/ZnO ^2%^ films exhibited the best water barrier properties, this ratio was fixed at 2%, and CH/ZnO/CO essential oil films were prepared with CO concentrations of 2–8% to enhance the films’ properties, which were subsequently characterized. As the CO concentration increased from 0% to 8.0%, CH/ZnO 2% film samples showed significant increases in thickness and EAB (%) by approximately 56.4% and 63.2%, respectively, while WS (%), MC (%), WVP, TS, and EM all decreased significantly by approximately 29.8%, 27.0%, 45.1%, 44.7%, and 47.8%, respectively (*p* < 0.05). These findings are attributed to the hydrophobic (apolar) nature of CO oil, which likely acts as a plasticizer within the polymer network, and the observed decrease in the two endothermic peak temperatures in the DSC values corresponds with the reduction in mechanical strength.

Analysis of light transmittance values indicated that higher CO oil concentrations decreased transmittance across the UV and visible spectra, increased opacity, and consequently improved the film’s UV-protective properties. Incorporation of nano ZnO and CO oil into CH-based films confers enhanced functional properties, including reduced WS (%), augmented antimicrobial efficacy, and diminished light transmittance, representing a sustainable, environmentally compliant, and waste-valorizing strategy with potential applicability as an alternative to petroleum-derived packaging materials. These results demonstrate that while CH/ZnO^2%^ films exhibit limited inherent antioxidant activity, the addition of CO markedly improves the radical-scavenging performance in a concentration-dependent manner, highlighting the potential of CH/ZnO^2%^/CO-based films as effective antioxidant packaging materials. Accordingly, future studies should systematically investigate the migration behavior of ZnO-containing films under realistic food-contact conditions to ensure compliance with EFSA and FDA regulatory limits, as well as include comprehensive antimicrobial testing to further evaluate the functional performance of the films for packaging applications.
